# Idiopathic Tapia Syndrome Following Routine Orotracheal Intubation in a Young Adult: A Case Report

**DOI:** 10.7759/cureus.102034

**Published:** 2026-01-21

**Authors:** Miguel A Pérez Malespín, Carlos A Soza Ruiz

**Affiliations:** 1 Faculty of Medical Sciences, Universidad Nacional Autónoma de Nicaragua (UNAN) Managua, Managua, NIC; 2 Department of Neurology, Hospital Alemán Nicaragüense, Managua, NIC

**Keywords:** dysarthria, dysphagia, endotracheal intubation, hypoglossal nerve injury, postoperative neurological complication, tapia syndrome, vagus nerve injury

## Abstract

Tapia syndrome is an extremely rare neurological complication affecting both the vagus (X) and hypoglossal (XII) nerves, usually as a result of mechanical trauma during endotracheal intubation. Associated symptoms can cause significant difficulties in swallowing and communication.

We present the case of a 20-year-old male with no relevant medical history who developed dysarthria, dysphagia, and rightward tongue deviation after routine intubation for an elective laparoscopic appendectomy. Neurological evaluation and electromyography showed no central or compressive lesions, confirming the clinical suspicion of post-intubation Tapia syndrome. Intensive speech therapy and close clinical follow-up resulted in complete recovery of lingual and vocal function within three months. From the patient’s perspective, the sudden onset of speech and swallowing difficulties caused significant anxiety and functional limitations, and the patient expressed satisfaction with the complete recovery achieved.

This case highlights the importance of early detection of post-anesthetic neurological complications, even after procedures considered low-risk, and underscores the effectiveness of timely multidisciplinary management.

## Introduction

Tapia syndrome is a rare cranial neuropathy characterized by the concurrent involvement of the vagus nerve (X) and the hypoglossal nerve (XII), most commonly associated with surgical or anesthetic procedures involving the head, neck, or thoracic region [1]. Clinically, it typically presents with dysarthria, dysphagia, and unilateral tongue deviation, and may be accompanied by ipsilateral vocal cord paralysis due to recurrent laryngeal nerve involvement [1,2]. Although uncommon, Tapia syndrome has been increasingly reported in perioperative and intensive care settings, highlighting its clinical relevance despite its low overall incidence [3-5].

The pathophysiology of Tapia syndrome is primarily attributed to mechanical compression, stretching, or ischemic injury of the affected cranial nerves at the level of the tongue base or within the retropharyngeal space [1,2]. Identified contributing factors include endotracheal intubation, excessive cuff pressure, prolonged intubation or tracheostomy, abrupt head and neck movements, posterior cervical spine surgery, and the use of large-caliber endotracheal tubes [2,4-6]. The occurrence of Tapia syndrome in previously healthy patients following apparently uncomplicated anesthetic procedures underscores the importance of early recognition and increased clinical vigilance in routine airway management [1,5,7].

## Case presentation

**Figure 1:**
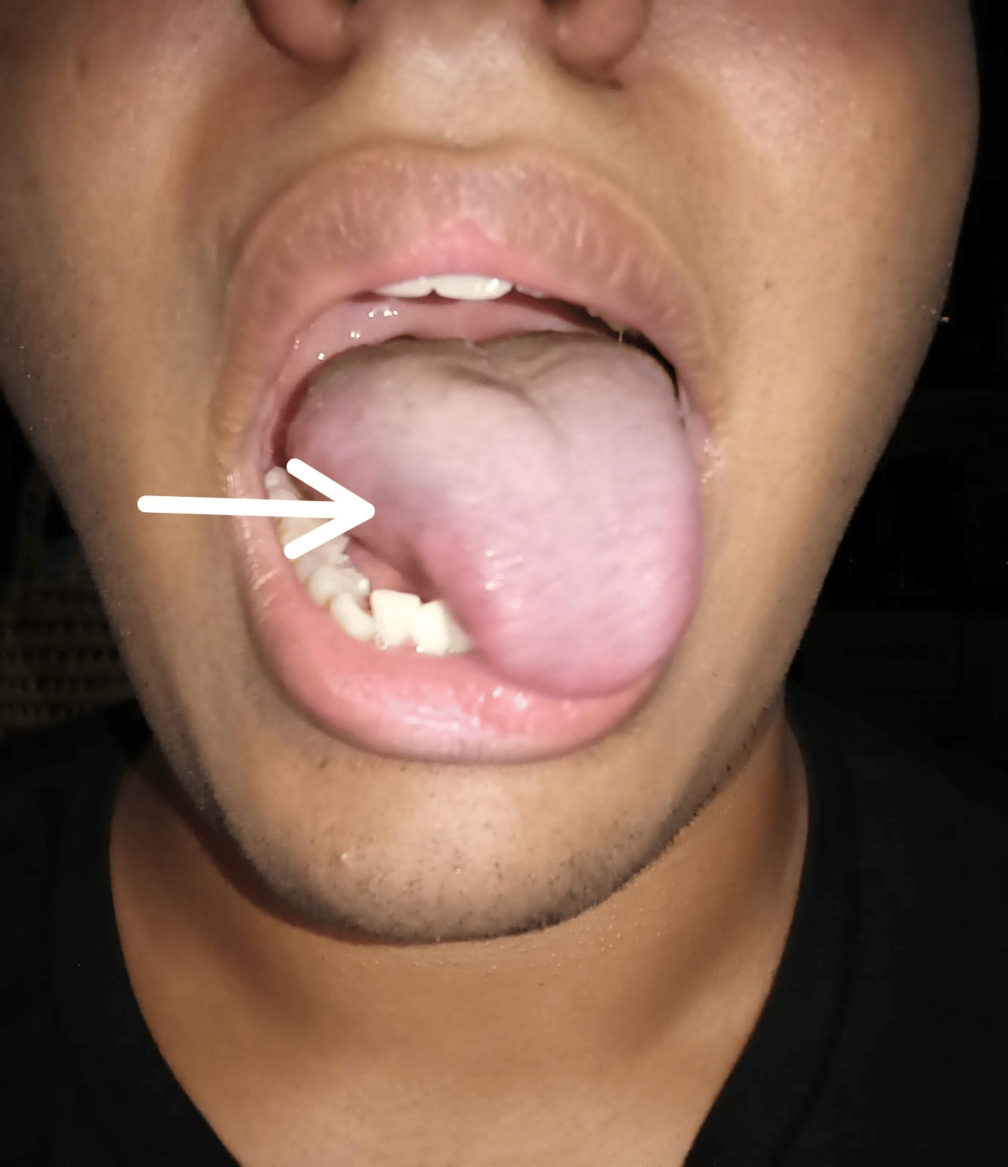
Rightward tongue deviation observed three hours after endotracheal intubation

A 20-year-old male with no relevant medical history underwent an elective laparoscopic appendectomy under general anesthesia. Orotracheal intubation was performed using a standard 7.0 mm tube, with the head and neck in a neutral position. The surgery lasted 90 minutes without intraoperative events. Immediately after emergence from anesthesia, the patient presented with dysarthria, dysphagia, rightward tongue deviation (Figure 1), and ipsilateral vocal cord paralysis detected via flexible laryngoscopy performed on postoperative day 2. The gag reflex was preserved, and no motor or sensory deficits were observed in the extremities, excluding significant central involvement. Electromyography of the tongue and vocal cords showed mild denervation of the X and XII cranial nerves, supporting the diagnosis without the need for imaging studies.

Management was conservative and included daily speech therapy and supervised swallowing exercises, complemented by weekly clinical follow-up. Progressive improvement in articulation and swallowing was observed during the first weeks, achieving complete recovery of lingual and vocal function by three months. No complications or recurrences were reported, and the patient resumed normal daily activities without functional limitations.

In this case, despite an uneventful intubation and surgery with no identifiable mechanical, positional, or anesthetic risk factors, the syndrome was considered idiopathic. Written informed consent was obtained from the patient for publication of this case report and accompanying clinical details.

## Discussion

Post-intubation Tapia syndrome is a rare neurological complication associated with airway manipulation, with fewer than 100 cases reported in the literature following orotracheal intubation for anesthesia. It typically presents with the characteristic triad of dysarthria, dysphagia, and unilateral tongue deviation after airway instrumentation. The underlying mechanism involves mechanical injury to the hypoglossal (XII) nerve and the recurrent laryngeal branch of the vagus (X) nerve along their extracranial course, usually caused by compression or stretching during intubation or prolonged ventilation [1,2]. Although most reported cases occur after complex head, neck, or thoracic procedures, recent reports demonstrate that Tapia syndrome can also develop following surgeries considered low risk, including elective abdominal procedures performed with standard intubation techniques. Conversely, the use of corticosteroids and other pharmacological interventions has been documented in some cases, but their efficacy is not definitively established [3]. The differential diagnosis includes brainstem ischemic stroke, skull base lesions, motor neuron disease, myasthenia gravis, and isolated hypoglossal or recurrent laryngeal nerve palsy. In this case, the absence of additional focal neurological deficits, preserved gag reflex, and supportive electromyographic findings favored a peripheral neuropathy related to airway manipulation rather than a central etiology [1,2]. Conservative management remains the mainstay of treatment, with most patients achieving favorable functional recovery. High clinical suspicion combined with early intervention and a multidisciplinary approach including anesthesiologists, neurologists, and speech therapists can improve outcomes and minimize associated morbidity, even in patients without classical risk factors [4-6]. Early initiation of speech and swallowing therapy has been consistently associated with improved outcomes and shorter recovery times [2,3,7]. Complementary approaches, such as electroacupuncture, have shown promising results for shorter recovery times, although further validation is required [3,7]. Although the syndrome developed after routine orotracheal intubation, the absence of identifiable precipitating factors or technical complications justified its classification as idiopathic.

## Conclusions

This case demonstrates that Tapia syndrome can occur after standard orotracheal intubation in young, otherwise healthy patients undergoing low-risk surgery. Early recognition of the characteristic clinical triad allowed diagnosis through clinical evaluation and electromyography without the need for imaging studies. Favorable outcomes with exclusively conservative management and early speech therapy highlight the effectiveness of timely functional interventions and underscore the importance of maintaining high clinical suspicion in the immediate postoperative period to optimize recovery and reduce associated morbidity.
